# Entomological Surveillance of *Aedes* Mosquitoes: Comparison of Different Collection Methods in an Endemic Area in RIO de Janeiro, Brazil

**DOI:** 10.3390/tropicalmed7070114

**Published:** 2022-06-22

**Authors:** Daniel Cardoso Portela Câmara, Claudia Torres Codeço, Tania Ayllón, Aline Araújo Nobre, Renata Campos Azevedo, Davis Fernandes Ferreira, Célio da Silva Pinel, Gláucio Pereira Rocha, Nildimar Alves Honório

**Affiliations:** 1 Laboratório de Mosquitos Transmissores de Hematozoários—LATHEMA, Instituto Oswaldo Cruz, Fundação Oswaldo Cruz, Rio de Janeiro 21040-900, Brazil; 2 Núcleo Operacional Sentinela de Mosquitos Vetores—Nosmove, Fundação Oswaldo Cruz, Rio de Janeiro 21040-900, Brazil; tasainat@gmail.com (T.A.); pinelcelio@gmail.com (C.d.S.P.); glaucioprocha@gmail.com (G.P.R.); 3 Programa de Computação Científica—PROCC, Fundação Oswaldo Cruz, Rio de Janeiro 21040-900, Brazil; codeco@fiocruz.br (C.T.C.); aline.nobre@fiocruz.br (A.A.N.); 4 Laboratório de Doenças Febris Agudas, Instituto Nacional de Infectologia Evandro Chagas, Fundação Oswaldo Cruz, Rio de Janeiro 21040-900, Brazil; 5 Instituto de Microbiologia Paulo de Góes, Universidade Federal do Rio de Janeiro—UFRJ, Rio de Janeiro 21941-901, Brazil; renatacampos@micro.ufrj.br (R.C.A.); davisf@micro.ufrj.br (D.F.F.)

**Keywords:** mosquito vectors, *Aedes*, entomological surveillance, trap comparison, adultrap, BG-Sentinel, CDC light trap, backpack aspiration

## Abstract

Using collection methods for *Aedes* adults as surveillance tools provides reliable indices and arbovirus detection possibilities. This study compared the effectiveness of different methods for collecting *Ae. aegypti* and *Ae. albopictus* and detecting arboviruses circulating in field-caught female specimens. Collection sites were defined in urban, peri-urban, and rural landscapes in two Brazilian cities. Collections were performed using Adultraps (ADT), BG-Sentinel (BGS), CDC-like traps (CDC), and indoor (ASP-I) and outdoor (ASP-O) aspiration during the rainy and dry seasons of 2015 and 2016. Generalized linear mixed models were used to model the effectiveness of each collection method. A total of 434 *Ae. aegypti* and 393 *Ae. albopictus* were collected. In total, 64 *Ae. aegypti* and sixteen *Ae. albopictus* female pools were tested for DENV, CHIKV, ZIKV, or YFV; none were positive. Positivity and density were linear at low densities (<1 specimen); thereafter, the relationship became non-linear. For *Ae. aegypti*, ADT and CDC were less effective, and ASP-I and ASP-O were as effective as BGS. For *Ae. albopictus*, all collection methods were less effective than BGS. This study highlights the need for an integrated surveillance method as an effective tool for monitoring *Aedes* vectors.

## 1. Introduction

Dengue, chikungunya, Zika, and yellow fever epidemics are important public health problems in several countries, particularly Brazil. The viruses that cause these diseases circulate in urban and peri-urban environments, where *Aedes aegypti* (Linnaeus, 1762) is considered the main vector and *Aedes albopictus* (Skuse, 1895) is a potential vector. All four viruses originated elsewhere and have been successfully introduced in the Americas [1,2,3,4]. *Aedes aegypti* is hypothesized to have been introduced to the Americas throughout the 15th to 17th centuries aboard slave ships [5,6]. After a massive eradication program in the 1940s and 1950s, *Ae. aegypti* was considered eradicated in Brazil, reinfesting it again in 1976 [7,8,9]. *Aedes albopictus* was introduced in the Americas during the 1980s, being detected in the US in 1985 and in Brazil in 1986 [10,11]. Both species are now widespread throughout the Americas [5].

Traditional surveillance of *Ae. aegypti* and *Ae. albopictus* is based on periodic household inspections [12,13]. Health agents search for the presence of containers bearing immature *Stegomyia*, which provide three widely used infestation indices: house (HI), container (CI), and Breteau (BI). Since it has become a convention that HI < 1% or BI < 5 were sufficient to prevent yellow fever transmission, these thresholds have been applied to dengue epidemics, with enormous criticism [13,14,15,16,17,18]. The use of traps as surveillance tools has long been proposed, providing qualitative indices (such as positivity per trap) and quantitative indices (such as density or the number of collected individuals per trap) [18,19,20]. Any given trap is subject to limitations in its sensitivity to collect mosquitoes of certain life stages or nutritional status or even mosquitoes of certain genera or subgenera [18,21]). Use of battery-powered aspirators for the surveillance of *Ae. aegypti* and *Ae. albopictus* has been very effective in in- and outdoor collections [22,23]. One advantage of this method is that it allows the active collection of both males and females from their resting sites, which allows for more precise data on the mosquito population in an area, such as richness, abundance, age structure, parity, and ovarian development [21,24].

Another advantage of using traps or automatic aspiration is the possibility of incorporating arbovirus surveillance into the entomological surveillance routine. The detection of infected mosquitoes collected in the field constitutes a useful early warning tool for arbovirus outbreak prediction or serotype introduction in endemic areas, as shown for dengue epidemics [23,25,26,27]. Furthermore, Zika virus (ZIKV) was detected circulating in field-collected *Ae. aegypti* mosquitoes, long before the first case of autochthonous ZIKV disease was reported in Rio de Janeiro [28]. Another important role of arbovirus surveillance is determining the prevalence of arboviruses and different serotypes in the community, as changes in the epidemiological profile are highly dynamic in endemic areas [29].

Here, we present the results of a two-year entomological and arbovirus surveillance for dengue, chikungunya, and Zika in two endemic cities in Brazil. We analyzed five different methods to capture adult *Ae. aegypti* and *Ae. albopictus*: Adultraps, BioGent-Sentinel, CDC-like traps (light and CO_2_), and automatic indoor and outdoor aspiration. This study aimed to compare five different collection methods and their effectiveness in capturing *Ae. aegypti* and *Ae. albopictus* in a heterogeneous landscape and to detect the most important arboviruses circulating in field-caught female mosquitoes.

## 2. Materials and Methods

### 2.1. Study Area

**Figure 1:**
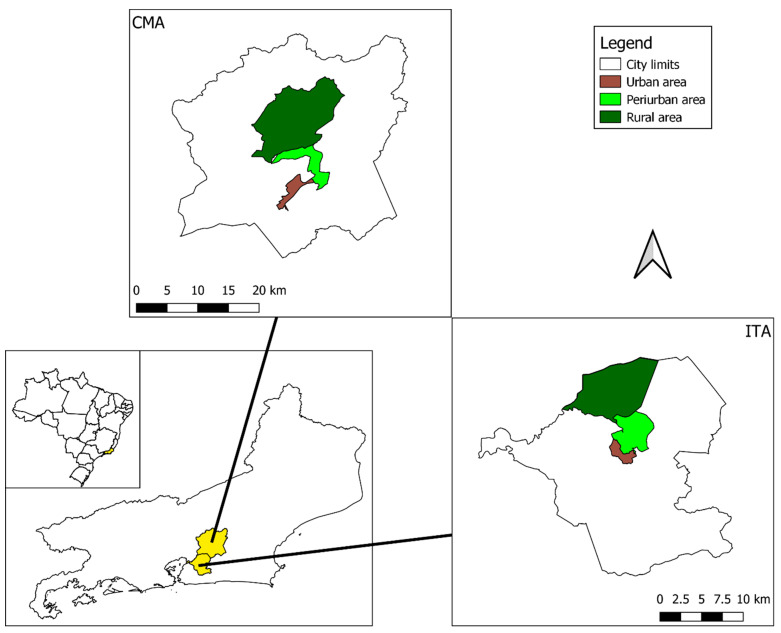
Map of the state of Rio de Janeiro showing the location of Cachoeiras de Macacu (CMA) and Itaboraí (ITA). Collection areas comprise one or more neighborhoods and are divided into urban (brown), peri-urban (light green), and rural (dark green).

**Figure 2:**
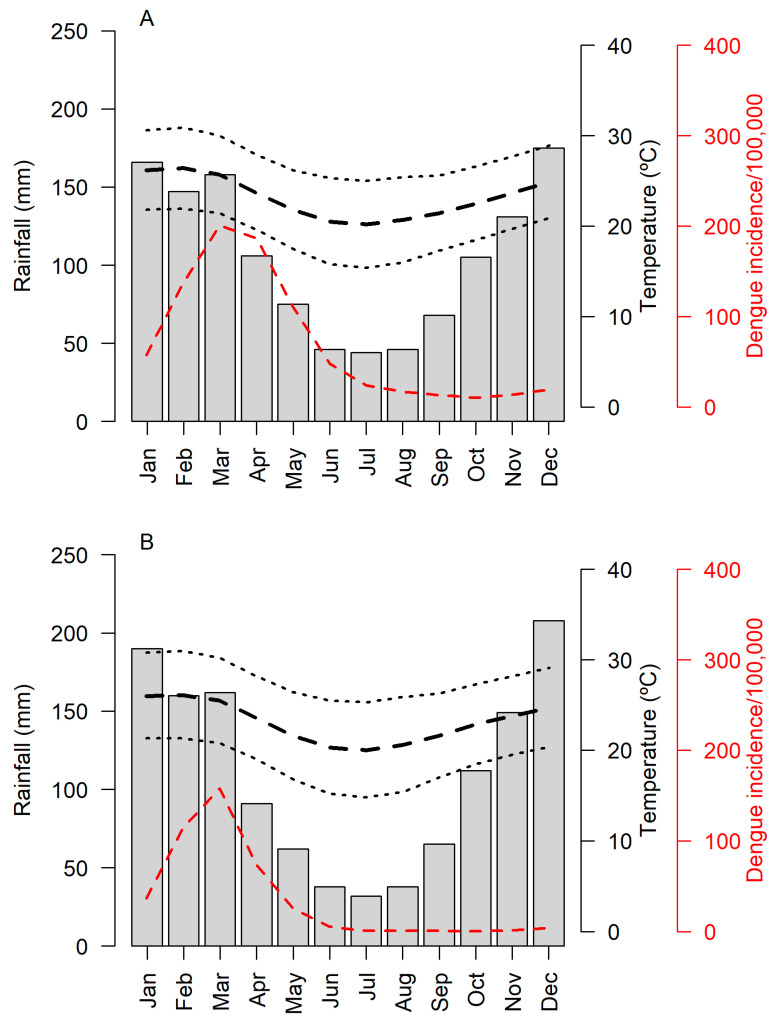
Climate and dengue transmission in (**A**) Itaboraí and (**B**) Cachoeiras de Macacu. Gray bars represent monthly accumulated rainfall. Solid and dashed black lines represent mean and minimum and maximum temperatures (°C). Red lines represent mean monthly dengue incidence from 2001–2019 (cases per 10,000 inhabitants) (Source: Sistema de Informação de Agravos de Notificação—SINAN).

Entomological collections were performed inside and in the peridomicile of human dwellings located in two adjacent cities in the state of Rio de Janeiro: Itaboraí (ITA, 22°44′40″ S, 42°51′34″ W, 17 m elevation) and Cachoeiras de Macacu (CMA, 22°27′45″ S, 42°39′11″ W, 54 m elevation) (Figure 1). The rainy season in the region occurs from December–March, with occasional floods (mean accumulated monthly rainfall, ITA: 44–175 mm, CMA: 32–208 mm), whereas the dry season extends from April–November. Temperature follows the same pattern as rain, with higher values during the wet season (mean monthly temperature, ITA: 15.4–30.9 °C; CMA: 14.8–31 °C). Dengue incidence has a delayed correlation with the wet season, with transmission rising in January and reaching a peak in March–April, a few months after the beginning of the wet season (mean monthly incidence from 2001–2019, ITA: 10.21–200.45 cases/100,000 inhabitants; CMA: 0.39–423.30 cases/100,000 inhabitants; Sistema de Informação de Agravos de Notificação—SINAN, data available at http://portalsinan.saude.gov.br/dadosepidemiologicos-sinan, accessed on 11 November 2021) (Figure 2). Human cases of yellow fever were reported in both cities in the 2017/2018 season, with one case reported in Itaboraí and three cases in Cachoeiras de Macacu (Secretaria de Estado de Saúde—SES/RJ, available at http://www.riocomsaude.rj.gov.br/Publico/MostrarArquivo.aspx?C=L4wqOoj4OVw%3d, accessed on 11 November 2021).

### 2.2. Mosquito Sampling

Three distinct areas were chosen in each city, based on sociodemographic and landscape profiles during field evaluations carried out with local health agents. These were classified as urban, peri-urban, or rural. In each area, 14 city blocks were randomly selected, and in each city block, one collection site was selected by convenience in the presence of local health agents. All collection sites were houses. At each collection site, a pair of mosquito traps were installed in the peridomicile near the house, chosen from Adultrap (Berdon, Curitiba, Brazil; hereafter ADT), BG-Sentinel (BioGents, Regensbourg, Germany; hereafter BGS), and CDC-like (Horst Armadilhas Ltda, São Paulo, Brazil; hereafter CDC). Trap pairing was random, and each collection point had a combination of either ADT + BGS, ADT + CDC, or BGS + CDC, totaling the use of nine ADT, nine BGS and ten CDC on each area according to the combinations. Backpack aspiration was performed at all 14 collection sites in each city both indoor (Horst Armadilhas Ltda, São Paulo, Brazil; hereafter ASP-I) and outdoor (Horst Armadilhas Ltda, São Paulo, Brazil; hereafter ASP-O). Collections took place during the rainy and dry seasons of 2015 and 2016. Each collection site was sampled once per period, totaling 4 collections per site per season [30].

**Adultrap (ADT):** The ADT is a trap designed to collect the gravid *Ae. aegypti* females searching for oviposition sites using water or water and hay infusions as attractants. ADT does not require batteries or electricity. Attracted females entered the trap through a hole in the top and became trapped in the interior chamber. Water and hay infusions remain confined inside a closed compartment inside the trap, which does not allow females to reach it and oviposit [31,32,33]. We installed ADTs with water and hay infusions in our study [34]. ADTs remained in the field for seven days.

**BG-Sentinel (BGS):** A BGS is a portable trap that consists of a collapsible bucket with an opening at the top. It requires a battery or electricity to power a fan, capturing host-seeking females that fly near the opening and trap them inside a catch bag. BGS uses an attractant (BG-Lure) that releases an artificial human skin odor to attract host-seeking females, mainly those with anthropophilic behavior [35,36,37]. We installed a BGS with BG-Lure attractants and a canister containing dry ice in our study. The BGS remained in the field for 24 h.

**CDC-like (CDC):** CDC traps are one of the most widely-used traps and consist of a portable trap with a battery-powered fan, which captures host-seeking females that fly near the opening at the top of the trap, sucking them into a catch bag at the bottom [38,39,40]. Installed CDC-like traps used in our study were equipped with standard light, UV light, and also a canister with dry ice. CDC traps remained in the field for 24 h [41].

**Backpack aspiration (ASP):** Battery-powered aspirators collect mosquitoes directly from their resting sites and are considered one of the most effective methods for collecting *Ae. aegypti* and other mosquitoes in domestic environments [36,42,43,44,45]. In our study, backpack aspiration was performed at each collection site, the day after collecting either BGS or CDC traps, since ADT remained 7 days in the field. Aspiration was performed for 20 min indoors (ASP-I) and outdoors (ASP-O).

All adult mosquitoes were transferred to plastic cages lined with cotton, filter paper, and silica gel with an oral suction tube and killed using dry ice (−70 °C). All samples were transported to the Núcleo Operacional Sentinela de Mosquitos Vetores, Nosmove/Fiocruz inside dry ice and protected inside the prepared cages. Species determination was performed by direct observation of morphological characteristics using a stereomicroscope, according to the taxonomic keys proposed by [46]). *Aedes aegypti* and *Ae. albopictus* females were classified as engorged or non-engorged based on the presence of digested blood in their abdomen.

### 2.3. Viral Detection

Pools of up to 10 engorged female mosquitoes were prepared and separated by species, date, collection method, and site. RNA was extracted from 140 µL of homogenized mosquito in 1 mL of Dulbecco’s modified Eagle’s medium (DMEM) supplemented with 3% fetal calf serum, 2.5 µg/mL amphotericin B, 500 U/mL penicillin, and 100 µg/mL streptomycin, using a kit specific for viral RNA extraction (QiaAmp Viral RNA Mini Kit, Qiagen, Valencia, CA, USA), according to the manufacturer’s protocol. Sample screening was performed by reverse transcription real-time PCR (RT-qPCR) using the QuantiTect Probe RT-PCR Kit (QIAGEN, Valencia, CA, USA) according to the manufacturer´s instructions. RT-qPCR protocols were used for ZIKV [47], CHIKV [48], DENV [49], and YF [50]. RT-qPCR was performed on a Thermo LightCycler^®^ 480 II instrument (BIOTECON Diagnostics, Postdam, Germany). PCR cycling conditions were adapted according to the enzyme kit used (ZIKV: 55 °C for 30 min, 95 °C for 10 min, followed by 45 cycles of 95 °C for 10 s and 60 °C for 45 s; DENV and CHIKV: 50 °C for 15 min, 95 °C for 10 min, followed by 45 cycles of 95 °C for 10 s and 60 °C for 40 s; YFV: 45 °C for 15 min, 95 °C for 2 min, followed by 45 cycles of 95 °C for 15 s and 60 °C for 30 s, and final 40 °C for 30 s).

### 2.4. Data Analysis

#### 2.4.1. Relationship between Positivity and Density Indices

We calculated two indices to measure the effectiveness of each collection method for both species: a positivity index (measured as the number of positive collections divided by the total number of collections at each collection site) and a density index (measured as the number of captured mosquitoes divided by the total number of collections at each collection site). Indices were calculated considering positive and negative collections (e.g., where a negative collection is defined as those where traps were installed and aspiration was performed, but no specimens were collected). Exploratory analysis was performed using locally weighted scatterplot smoothing models (LOESS) to visually identify non-linear relationships between both indices. After assessing the nonlinearity of the relationships, we used generalized additive models (GAM) [51] to verify the possible saturation between positivity and density indices. A model was constructed for each species (*Ae. aegypti* and *Ae. albopictus*) using the positivity index for each collection method (ADT, BGS, CDC, ASP-I, or ASP-O) as response variables and a smooth term for the corresponding density index as the explanatory variable. The models were constructed using a Gaussian distribution. We compared each non-linear model with a linear model and selected the best model using Akaike information criterion values [51].

#### 2.4.2. Generalized Linear Mixed Models

We used generalized linear mixed models (GLMM) from a Bayesian perspective using Markov chain Monte Carlo (MCMC) to quantify the abundance of *Ae. aegypti* and *Ae. albopictus* collected using each collection method in each landscape and city. The outcome variable was the number of adults collected per mosquito species at each collection site (*Ae. aegypti* or *Ae. albopictus*). We used a negative binomial distribution due to super dispersion [52]. The fixed effects were landscape (a categorical variable with three levels: urban, peri-urban, and rural), season (a categorical variable with two levels: dry and wet), and collection method (categorical with five levels: Adultrap, BG-Sentinel, CDC, Indoor Aspiration, and Outdoor Aspiration). We chose BGS as the baseline level for the collection method variable because of its known efficacy in trapping *Ae. aegypti* and *Ae. albopictus* in domestic and peridomestic environments [53,54]. The baseline level for landscape was “urban” (allowing us to compare peri-urban and rural with it); for season as “wet” (allowing us to compare the dry season with the wet season). Thus, results are exhibited as a comparison and discussed accordingly. The logarithm of the number of successfully retrieved traps and aspirations performed at each collection point was included as the model offset. Traps that exhibited a failure were discarded from the analysis (such as not having battery when being retrieved, traps that were lost, collections sites that could not be aspirated by any reason, etc.). At the end of the study, a total of twelve observations were excluded due to failure. We included the collection site as a random effect because of the repeated measures nature of the data [52]. Models were constructed using the RStan (Stan Development Team, 2022; Version 2.21.5, https://mc-stan.org/, accessed on 11 November 2021) and RStanarm (Goodrich et al., 2022; Version 2.21.3, https://mc-stan.org/rstanarm/, accessed on 11 November 2021) packages in R software (R Core Team, 2022; https://www.R-project.org/, accessed on 11 November 2021). Model validation and selection were performed via leave-one-out (LOO) and LOO information Criterion (LOOIC) using the loo package [55].

## 3. Results

### 3.1. General Results and Viral Detection

**Figure 3:**
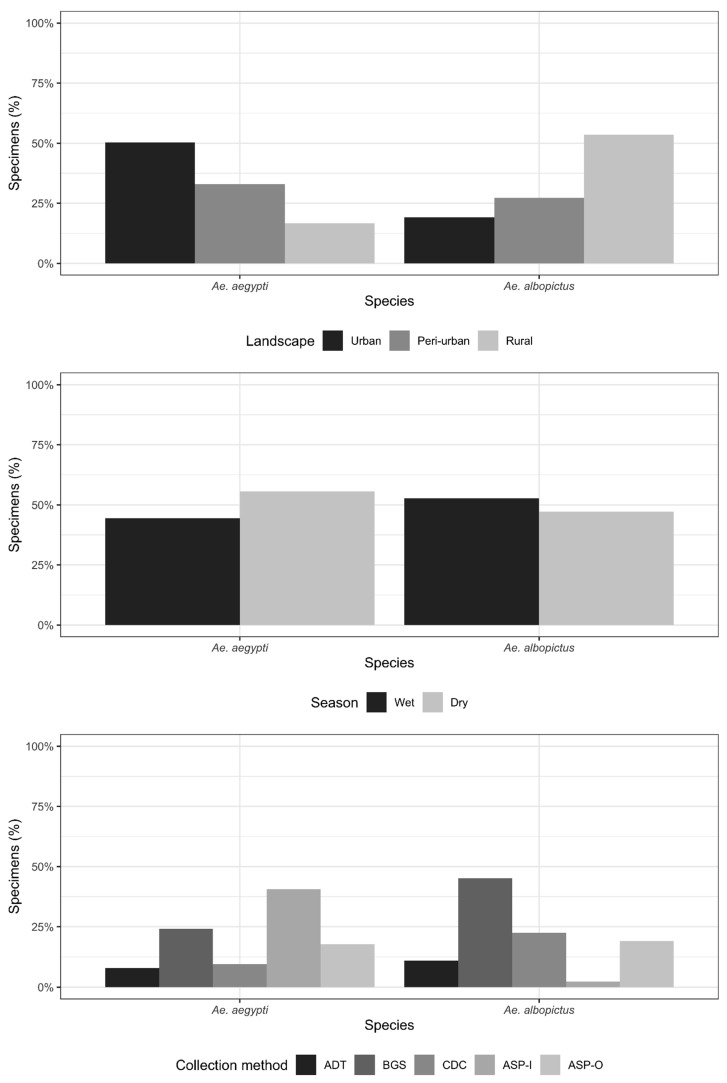
Exploratory analysis of the total number of *Ae. aegypti* and *Ae. albopictus* specimens collected in the study. Top: percentage of *Ae. aegypti* and *Ae. albopictus* specimens collected according to landscapes (urban, peri-urban and rural). Middle: percentage of *Ae. aegypti* and *Ae. albopictus* specimens collected according to season (wet and dry). Bottom: percentage of *Ae. aegypti* and *Ae. albopictus* specimens collected according to collection method (ADT, BGS, CDC, ASP-I and ASP-O).

A total of 434 *Ae. aegypti* (191 males and 243 females) and 393 *Ae. albopictus* (79 males and 314 females) specimens were collected during the study period (Table 1). Exploratory analysis revealed a higher number of *Ae. aegypti* in the urban landscape (50, 3%), with progressively less specimens collected in the peri-urban and rural landscapes (33.0% and 16.6%, respectively). The pattern was opposed for *Ae. albopictus*, with fewer specimens collected in the urban landscape (19.1%), and progressively more individuals in the peri-urban and rural landscape (27.3% and 53.6%, respectively). More *Ae. aegypti* specimens were found in the dry season (55.6%), whereas more *Ae. albopictus* specimens were found in the wet season (52.8%). A total of 40.5% of the specimens of *Ae. aegypti* were collected using ASP-I, whereas the majority of Ae. albopictus was collected using BGS (45.2%). ADT and CDC collected the lowest number of *Ae. aegypti* specimens (7.9% and 9.5%, respectively), whereas ADT and ASP-I collected the lowest number of *Ae. albopictus* (11.0% and 2.3%, respectively) (Figure 3). We observed a significant difference in the number of engorged *Ae. aegypti* per landscape and collection method (Fisher’s exact test *p*-value = 0.0039) but not for *Ae. albopictus* (Fisher’s exact test: 0.2414). A total of 64 and 16 pools of *Ae. aegypti* and *Ae. albopictus* engorged females were tested for the presence of DENV, CHIKV, ZIKV, or YFV RNA using real-time RT-PCR, and none showed positive results.

### 3.2. Relationship between Positivity and Density Indices

**Figure 4:**
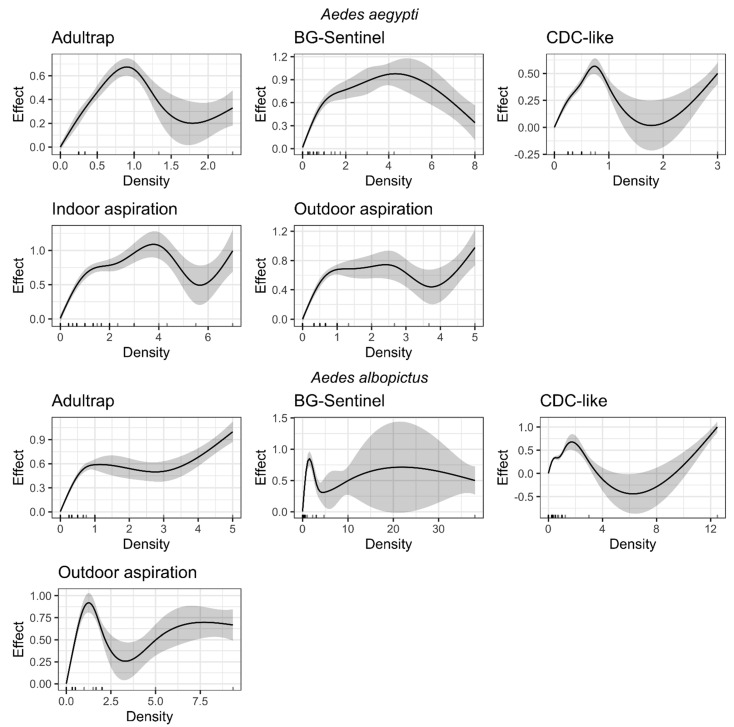
Estimated smoothing curves for *Ae. aegypti* (top) and *Ae. albopictus* (bottom). The solid line represents the smoother function; the grey area represent 95% confidence intervals. ADT = Adultrap, BGS = BG-Sentinel, CDC = CDC light trap, ASP-I = Indoor aspiration, ASP-O = Outdoor aspiration. Dotted red lines are placed at zero to facilitate visual interpretation. It was impossible to fit an ASP-I model for *Ae. albopictus* because of the low number of observations. *x*-axis represent the density indexes for each trap and each species; *y*-axis represent the effect of the density index on the positivity index for each trap and each species.

Figure 4 shows the smoothing curves of the GAM models of positivity versus density indices for the five collection methods for both *Ae. aegypti* and *Ae. albopictus*. For both species, the relationship between the indices was significant and non-linear, with the positivity index peaking and plateauing at intermediate values of the density index. The total number of *Ae. aegypti* and *Ae. albopictus* was aggregated to generate the positivity and density indexes for each collection site for each species (Supplementary Material Table S1).

For *Ae. aegypti*, there was an overall linear relationship between both indices when density was lower than one adult per collection method. Nonlinearity was observed when the number of adults per collection method increased. The ADT positivity index peaked at ca. 0.7 when the density index was ca. 1 and stabilized after this value. BGS positivity peaked at ca. 0.9 with a density of ca. four mosquitos per trap. CDC positivity peaked at ca. 0.6 with a density lower than one mosquito per trap. The ASP-I positivity peaked at ca. 1 when the indoor density was at ca. four mosquitos. Finally, ASP-O positivity peaked at ca. 0.75, with an outdoor density lower than 2 mosquitoes, but plateaus when the outdoor density was 1–3 mosquitos.

For *Ae. albopictus*, the ADT positivity index peaked at ca. 0.6. The density index peaked at ca. one and plateaued thereafter. The BGS peaked at ca. 0.75 with a density of ca. two mosquitoes per trap and plateaued at this positivity index, which decreased when the number of mosquitoes per trap reached four individuals. CDC positivity peaked at ca. 0.7 when the density was ca. two mosquitoes per trap. ASP-I did not provide reliable information because of the low number of observations, and it was not possible to run a GAM. ASP-O had a positivity of ca. 0.9 when the outdoor density was ca. one mosquito and decreased thereafter (Figure 4).

### 3.3. GLMM for Aedes Aegypti Abundance

The GLMM for Aedes aegypti results, while controlling for all other variables, did not show significant differences in the dry period when compared with the wet period, and the average number of collected Ae. aegypti decreased in the peri-urban and rural landscapes compared to the urban landscapes (a mean decrease of 44.12% and 66.34%, respectively). Collections using ADT and CDC resulted in a significantly lower average number of Ae. aegypti compared to BGS (a mean decrease of 65.32% and 55.34% in the number of specimens, respectively). However, ASP-I and ASP-O showed no significant differences when compared with BGS, meaning that on average, the three collection methods yielded the same number of specimens (Table 2).

### 3.4. GLMM for Aedes Albopictus Abundance

For Ae. albopictus, the model results showed that while controlling for all other variables, the average abundance of specimens was significantly lower (56.53%) in the dry season when compared to the wet season. We did not find a significant difference in the peri-urban landscape when compared to the urban landscape. However, the average abundance of Ae. albopictus specimens was 333.01% higher in the rural when comparing with the urban landscape. The average number of specimens of all collection methods were significantly lower (77.37%, 56.26%, 95.86% and 63.43% for ADT, CDC, ASP-I and ASP-O, respectively) when comparing with the BGS (Table 2).

## 4. Discussion

This study compared five collection methods and their effectiveness in capturing *Ae. aegypti* and *Ae. albopictus* in a heterogeneous landscape in Rio de Janeiro and detecting the most important arboviruses that circulate in field-caught females. Our results showed that the relationship between trap positivity and density index was non-linear. For *Ae. aegypti*, BGS and ASP-I had the highest positivity indices, with the BGS peaking at ca. 0.9 with a density of four and the former peaking at 1 also with a density of four mosquitos. However, the GLMM results for *Ae. aegypti* showed no significant difference when comparing ASP-I with BGS (baseline level), suggesting that both collection methods yielded similar results. These results show that BGS can be used by health agents when a premise is closed, otherwise impossible to enter, or if there are no automatic aspirators. We propose that these results were achieved because most of the productive containers for *Ae. aegypti* were located outdoors near houses, and a trained team could find resting adults nearby [56,57,58]. A backpack aspirator is a reliable tool for *Ae. aegypti* and *Ae. albopictus* surveillance, providing valuable information on biological traits, such as parity, survival, and physiological stages in endemic areas [21,24,42,59], while also being very sensitive in detecting preferred resting places for females [60,61]. Other studies using backpack aspirators described the distribution of *Ae. aegypti* and *Ae. albopictus* in endemic areas, with the former species being highly associated with urbanized areas and the latter with rural areas, similar to findings in the present study [22]. Our GLMM results showed a clear association between *Ae. aegypti* and the urban landscape, and *Ae. albopictus* and rural landscapes.

For *Ae. albopictus*, ASP-O, and BGS had the highest positivity indices, with the latter peaking at ca. 0.9, the former at 0.75, and a density of ca. two mosquitoes. This might be related to this vector preference for peridomicile areas, as observed under field conditions [22,62,63]. Despite the higher positivity rate for *Ae. albopictus* in the peridomicile, the GLMM results for this species showed no significant difference when comparing all collection methods with BGS. BGS traps have been successfully used in *Ae. aegypti* and *Ae. albopictus* surveillance, catching males and females in different physiological stages [35,64]. A mass-trapping study using BGS was promising for *Ae. aegypti* control, with a reduction in dengue cases in premises monitored by the trap, although this difference was not significant [65]. For *Ae. albopictus*, our results agree with those of other studies that indicate BGS as an effective tool for *Ae. albopictus* surveillance in North America [66,67], Italy [68,69], and Australia [70], where it showed good sensitivity for the detection of new mosquito species. However, of the ~675,000 mosquitoes caught in a mass trapping study using only BGS elsewhere in Brazil, less than 0.1% were *Ae. albopictus* [65]. Our results may indicate the use of BGS in areas with known *Ae. albopictus* infestation. Further investigations should be conducted to evaluate the potential role of this species in arbovirus transmission to humans [71].

In a multicenter study performed in Brazil, the authors measured four different traps and compared their effectiveness in larval surveys [13]. In that study, using Adultraps, BG-Sentinel, MosquiTRAPs, and ovitraps positivity indices were significantly more sensitive than using the house index for detecting and measuring *Ae. aegypti* infestation level. These authors stated that trap positivity indices could be used as a proxy for the density index because the first consistently plateaued when densities were high [13]. Results regarding the effectiveness of ADT in monitoring *Ae. aegypti* under field conditions are contrasting. In the original study where ADT was presented, a field trial was undertaken to test the trap in 120 houses in an area with an HI of 1.5%. The authors concluded that the trap captured *Ae. Aegypti*, but no information was provided on the number of positive traps or adult density per trap, with results reporting only 24 females captured after 24 h of monitoring [31]. A subsequent study reported the results of a trial comparing the indoor and outdoor placement of ADT and backpack aspiration, concluding that ADT was more effective than aspiration when placed outdoors, but not indoors [72], in contrast to the results of our study. The results of a multicenter study showed that ADT never exceeded a positivity index of 0.2, despite detecting seasonal fluctuations in population abundance [13]. The positivity in our study was ca. 0.7 *Ae. aegypti* and 0.6 for *Ae. albopictus*. In the same cited study, ADT sensitivity increased when the trap examination changed from 24 h to 96 h. Our study used a seven-day protocol, which might explain the higher positivity index. The GLMM results corroborate these observations, showing a significantly lower effect when compared with BGS for both species. Further studies are needed to evaluate the effectiveness of ADT for mosquito vector surveillance in heterogeneous endemic areas. The CDC-like traps used in our study were the least effective collection method, as indicated by the low positivity in the GAM results for *Ae. aegypti* (ca. 0.6) and *Ae. albopictus* (ca. 0.7) and by the significantly lower average number of mosquitoes collected compared with BGS in both GLMMs. These results are in agreement with those of other studies in the literature, where CDC tends to be one of the least effective methods for *Ae. aegypti* and *Ae. albopictus* surveillance compared with other traps [39,73,74].

Our results should take into consideration some adaptations that we have done in the three traps used. As mentioned previously, the original ADT trapping protocol proposed by the authors mentions a 24-h period [31], which was further expanded to 96 h in a multicentric study [13]. In our study, we have used a 7-day protocol and also added hay infusion to the water due to its known attractant properties to *Ae. aegypti* and *Ae. albopictus* females [13,19,34]. Although another study pointed to the lack of statistical significance when comparing ADT with and without hay infusion [75], a simple modification of the trap seems to increase its sensitivity [76], although this was not done in our study. Our BGS used BG-Lure and CO_2_, whereas our CDC-like traps also used CO_2_. Carbon dioxide is a known and important attractant for mosquitoes, which is an indication of the presence of hosts [77,78,79,80]. Other studies showed that using CO_2_ enhanced the efficacy of BGS to collect *Ae. aegypti* by increasing the positivity rate [81] and also *Ae. albopictus* [82], despite evidence that there is no statistical difference when comparing BGS with BG-Lure to BGS with CO_2_ in Florida [83]. In French Polynesia, a study showed that CDC traps collected significantly higher numbers of *Ae. aegypti* when used with CO_2_ [84]. Our adaptations were made in order to increase the odds of finding both species vector (presence) and also to increase their abundance. Further studies comparing these traps, with and without modifications, are necessary in heterogeneous and endemic areas for arboviruses.

In Brazil, current national *Aedes* control programs do not focus on adults to measure the infestation levels of *Ae. aegypti* and *Ae. albopictus*, rather, efforts target larval assays (Larval Index Rapid Assay for *Aedes aegypti*, LIR*Aa*). Despite the initial use of traditional larval indices such as a house, container, and Breteau to prevent yellow fever transmission, these and other indices have been routinely applied to dengue surveillance [17]. Criticism is mainly directed at shortcomings concerning the object of measurement of the indices: CI gives information about the proportion of positive containers in an area, without taking into account the number of immatures or houses; HI provides information about the proportion of infested houses with at least one immature *Ae. aegypti*, which does not indicate the number of positive containers per positive house [85]; and the BI, which combines the number of positive containers per 100 houses [86]. Moreover, these commonly used indices fail to report the adult mosquito productivity in inspected containers or houses, focusing only on the highly density-fluctuating larval stage [15,87]. As female adult mosquitoes are responsible for pathogen transmission to humans, any entomological surveillance procedure should produce estimates that reflect the female adult population in any given area [18].

One advantage of oriented entomo-virological surveillance in adult mosquitoes is the possibility of monitoring arbovirus introduction or changes in dengue serotype circulation. Our study did not have positive molecular results for the main arboviruses circulating in the study area: the four DENV serotypes, CHIKV, ZIKV, and YFV. We believe that this might be attributed to the relatively low number of collected mosquitoes in our study in comparison with estimates from the literature; for example, a minimum of ~700 mosquitos are required for an arbovirus detection probability of 0.5, whereas ~1600 and ~2300 are needed for detection probabilities of 0.8 and 0.9, respectively [88,89]. However, several studies have shown virus serotype detection in field-collected adult mosquitoes as a surveillance tool to support health services in Brazil [23,90,91,92] and elsewhere [25,93,94].

In Brazil, a patient-based surveillance study for dengue virus in *Ae. aegypti* detected DENV-1 and DENV-2 during an epidemic of DENV-3 in Recife [27]. Another study reported the circulation of DENV-4 in mosquitoes and humans during an epidemic of DENV-1 in Rio de Janeiro, one year after the introduction of DENV-4 in the country [95]. Another patient-based arbovirus surveillance in mosquito vectors detected ZIKV circulation in *Ae. aegypti* collected in an area endemic for arboviruses before the first autochthonous case was confirmed in human patients in Rio de Janeiro city [28]. In a study performed in Northeast Brazil, where entomo-virological surveillance monitoring was performed after risk stratification evaluation in Natal, RN, seven pools of male and female *Ae. aegypti* collected in public schools, health units, junkyards, recycling points, and residential premises were positive for DENV-3, reinforcing the need for continuous virological surveillance of *Aedes* mosquitoes [23]. In Mexico, a study showed the presence of ZIKV in the salivary glands of wild-caught female mosquitoes from five different species: *Ae. aegypti*, *Aedes vexans* (Meigen, 1830), *Culex quinquefasciatus* (Say, 1823), *Culex coronator* (Dyar and Knab, 1906) *and Culex tarsalis* (Coquillett, 1896), which may change the paradigm of ZIKV control to include the surveillance of other species [96].

In our study, GLMM showed that *Ae. aegypti* lack of variation in its density across the dry and wet seasons, with higher density in the urban landscape and progressively lower densities in suburban and rural landscapes. This was contrasted by the GLMM results for *Ae. albopictus*, which showed a decrease in density during the dry season, but not variation of densities when comparing urban and periurban environments. Contrary to *Ae. aegypti*, *Ae. albopictus* showed a significant increase in density in the rural landscape. The stability in *Ae. aegypti* could be related to its habitat preference for domestic environments. The relatively protected environment provided by housing may favor stable year-long breeding site availability during wet and dry seasons [22,97,98]. This result was contrasted by the decline of *Ae. albopictus* during the dry season. This might be related to the highly endophilic nature of Ae. aegypti and the more exophilic behavior of *Ae. albopictus*. Similar patterns have been found in Brazil [22], Trinidad [99], Panama [100], Sri Lanka [98]. Such preference to use human-stored water as fallback larval sites during dry seasons, is hypothesized to have initiated the domestic evolution of *Ae. aegypti* [101], and might be a factor already happening to *Ae. albopictus* in Rio de Janeiro [45]. Additionally, competition between both species is documented under field conditions [102,103], which might shape the distribution and abundance of both species in a heterogeneous landscape across different seasons. It is known that the drying of containers is more detrimental to *Ae. albopictus* than to *Ae. aegypti*, with several studies showing higher resistance to desiccation during the egg phase for *Ae. aegypti* when compared to Ae. albopictus [104,105,106]. This pattern of spatial and temporal distribution was also observed in a field study performed in Rio de Janeiro, Brazil, which shares many of its landscape and socioeconomic characteristics with Itaboraí and Cachoeiras de Macacu cities in our study [19]. In this study, the authors found no statistically significant negative correlation for any of the periods in the oviposition of *Ae. aegypti* and *Ae. albopictus* in a transition zone between densely urbanized and forested areas inside Rio de Janeiro. However, another study showed no significant differences in the abundances of *Ae. aegypti* and *Ae. albopictus* in southern Florida when comparing the wet and dry seasons, probably due to the greater abundance of *Ae. albopictus* in the area and the great availability of anthropogenic water sources around households during the dry season [107]. Finally, *Ae. albopictus* was found in high numbers in the rural landscape (more than twice the number of specimens in the peri-urban landscape, and almost five times the number of specimens in the urban landscape). Such pattern is in agreement with other findings in Brazil, that showed high densities of *Ae. albopictus* in peri-urban and rural areas, whereas in highly urbanized areas it is commonly found in close association to vegetated areas [19,22,103,108,109].

In our study, backpack aspiration (both indoors and outdoors) and BGS were equally effective for *Ae. aegypt* collection, and BGS was the best method for *Ae. albopictus* collection. Our study was performed in two cities, reflecting different heterogeneous landscapes where both vectors are present. Dengue is already endemic to the areas, and recent outbreaks were reported for chikungunya and Zika fevers. Arbovirus detection in field-caught mosquitoes is an important tool that should be integrated into routine surveillance, including equally effective methods for *Ae. aegypt* and *Ae. albopictus* collection. In addition, longitudinal studies should be undertaken to ensure that the probability of arbovirus detection increases. Routine arbovirus surveillance based on field collections of female mosquitoes, performed at specific times, as pointed out by the local epidemiological context of each city, could significantly enhance health authorities’ ability to respond to outbreaks and epidemics.

## Figures and Tables

**Table 1:** Total number of *Ae. aegypti* and *Ae. albopictus* specimens collected by each collection method in three different landscapes. The numbers presented are the total number of collected mosquitos/the total number of females (engorged females).

		*Aedes Aegypti*			*Aedes Albopictus*	
Method	Urban	Peri-Urban	Rural	Urban	Peri-Urban	Rural
ADT	19/19 (2)	11/10 (1)	5/5 (0)	8/7 (0)	25/25 (0)	10/10 (2)
BGS	78/36 (3)	10/6 (3)	18/11 (4)	22/21 (0)	46/43 (2)	109/84 (0)
CDC	15/11 (0)	5/3 (0)	22/17 (0)	27/26 (0)	19/19 (0)	43/42 (0)
ASP-I	81/42 (42)	85/44 (34)	12/7 (6)	4/2 (2)	3/3 (3)	2/2 (2)
ASP-O	28/9 (6)	34/23 (17)	11/0 (0)	14/5 (1)	15/9 (4)	46/16 (0)
Total	221/117 (53)	145/86 (55)	68/40 (10)	75/61 (3)	108/99 (9)	210/156 (4)

**Table 2:** Estimates, credibility interval (CI95%), and Standard Deviation of the GLMMs for *Ae. aegypti* and *Ae. albopictus*. Bold entries indicate statistical significance.

Effects	Estimates	*Aedes Aegypti*			*Aedes Albopictus*		
		Mean	CI95	SD	Mean	CI95	SD
Fixed	Intercept	**−1.653**	**(−2.147, −1.156)**	**0.250**	**−1.689**	**(−2.351, −0.909)**	**0.370**
	Period: Dry	−0.094	(−0.456, 0.279)	0.187	**−0.827**	**(−1.456, −0.219)**	**0.318**
	Landscape: Periurban	**−0.570**	**(−0.992, −0.140)**	**0.217**	0.562	(−0.141, 1.266)	0.358
	Landscape: Rural	**−1.069**	**(−1.510, −0.628)**	**0.225**	**1.189**	**(0.413, 1.960)**	**0.383**
	ADT	**−1.055**	**(−1.637, −0.453)**	**0.304**	**−1.471**	**(−2.349, −0.673)**	**0.421**
	CDC	**−0.803**	**(−1.412, −0.200)**	**0.306**	**−0.829**	**(−1.630, −0.066)**	**0.406**
	ASP-I	0.471	(−0.046, 1.000)	0.264	**−3.185**	**(−4.339, −2.132)**	**0.561**
	ASP-O	−0.273	(−0.803, 0.263)	0.278	**−1.003**	**(−1.867, −0.222)**	**0.411**
Random	Site	0.179	(0.014, 0.479)	0.121	0.016	(0, 0.114)	0.035

## Supplementary Materials

The following supporting information can be downloaded at: https://www.mdpi.com/article/10.3390/tropicalmed7070114/s1, Table S1: positivity and density indexes for each collection site for each species.
